# Parental Migration and the Social and Mental Well-Being Challenges among Indonesian Left-Behind Children: A Qualitative Study

**DOI:** 10.3390/ijerph21060793

**Published:** 2024-06-18

**Authors:** Nelsensius Klau Fauk, Alfonsa Liquory Seran, Paul Aylward, Lillian Mwanri, Paul Russell Ward

**Affiliations:** 1 Centre for Public Health, Equity and Human Flourishing, Torrens University Australia, 88 Wakefield St, Adelaide, SA 5000, Australia; paul.aylward@torrens.edu.au (P.A.); lillian.mwanri@torrens.edu.au (L.M.); 2 Atapupu Public Health Centre, Health Department of Belu District, Atambua Belu 85752, Indonesia; liquory.seran@yahoo.com

**Keywords:** parental migration, social and mental well-being, left behind children/adolescents, Indonesia

## Abstract

Parental labour migration, of either one or both parents, has been associated with various challenges among left-behind children (LBC). However, there is a limited understanding of the LBC’s own views and experiences of social and mental well-being and how the new daily life circumstances they encounter following their parents’ migration impact them. This study aimed to understand the influence of parental migration and its aftermath on the social and mental well-being of adolescents (referred to as LBC) in two rural districts in Indonesia. Employing a qualitative design, data were collected through individual in-depth interviews with LBC (*n* = 24) aged 14 to 18 years, recruited using the snowball sampling technique. Data were thematically analysed, guided by a qualitative data analysis framework. The findings showed that parental migration negatively impacted the social well-being of LBC. This impact was reflected in negative labelling from friends and changes in familial roles which influenced their social interactions and activities with peers. Parental migration was also associated with challenges to the mental well-being of LBC. These manifested in the LBC experiencing fractured emotional bonds, leading to negative emotions, including stress, anxiety, sadness, depression, frustration, loss of motivation, and self-imposed isolation, which were associated with their parents’ migration and abrupt disruptions in parent–child communication. The transition to new life situations with caregivers after parental migration and the dynamics within the caregivers’ households were additional factors that detrimentally affected their mental well-being. Unmet basic needs and educational needs due to financial hardships faced by mothers and caregivers further exacerbated mental health challenges for the children. The findings indicate the importance and improvement of policies and interventions in Indonesia (e.g., counselling services, non-cash food assistance, family hope program, direct cash assistance) that cover and address the diverse needs of mothers or caregivers and the LBC.

## 1. Introduction

Migration, mostly from low- and middle-income countries (LMICs) to developed or high-income countries, is a global phenomenon [1,2]. According to the World Migration Report, there were an estimated 281 million international migrants globally in 2020, with around 169 million identified as international migrant workers [3,4]. International migrant workers are individuals who originate from one country, typically LMICs, and relocate to another country for employment purposes, with no intention of permanent settlement in the host country [3,5]. Parental migration to other places within a country or overseas in search of employment opportunities is part of labour migration. Parental migration has led to millions of left-behind children (LBC) throughout the world living and growing up experiencing prolonged separation from their parents and being cared for by other members of extended families such as grandparents, aunties, and uncles [6,7,8,9]. The United Nations International Children’s Emergency Fund (UNICEF) used the phrase ‘left-behind children’ to refer to children who remain in their home countries or countries of habitual residence when one or both of their parents or adults responsible for them migrate internally or to another country [10]. 

Studies worldwide have shown that, besides positive economic impacts or increased household incomes through remittances that also support LBC’s education [11,12,13], parental migration negatively influences the lives of children left behind [4,13,14]. Left-behind children, especially those with both parents migrating, are reported to have more emotional problems and are more likely to experience poor psychological well-being compared to their counterparts [15,16,17,18,19]. Studies in Asian countries such as China, Vietnam, Nepal, Thailand, and the Philippines have reported that compared to children with non-migrating parents, LBC have higher levels of depression, anxiety, loneliness, and lower levels of self-esteem, and have been reported to experience reduced happiness [13,16,18,19,20,21,22]. Similarly, studies from across the world including African countries [23,24], Central America, and North America have also reported that children with migrating parents have poorer mental health outcomes (with symptoms of depression, anxiety, and poor well-being) compared to children with non-migrating parents [15,25]. In addition to poor mental health outcomes related to separation, previous studies have reported that parental absence due to migration leads to inadequate care and support which are recognised as risk factors for depression and anxiety for LBC [14,26]. Moreover, low academic performance, staying up late, having difficulty falling asleep, and experiencing physical abuse have been reported as factors associated with challenging the mental health of LBC [14,26]. 

Globally, despite the association of parental labour migration with poor psychological well-being of the LBC, there remains a limited understanding of social well-being and child–parent separation-related risk factors for the children’s mental well-being. There is also a paucity of evidence on the children’s own views and experiences of how the new daily life situations they encounter following parental migration influence their social and mental well-being [15,27]. Additionally, previous studies on the topic have mainly focused on examining the significance of the association of parental labour migration with poor mental health well-being of the LBC, with a paucity of in-depth qualitative research seeking to explore and understand the mental health challenges that these children face [14,15,17,27,28]. Indonesia is a significant contributor to the international migrant workforce, with over four million Indonesian labour migrants employed in more than twenty-five countries globally [29]. This figure is expected to rise steadily, given the notable increase in the annual outbound migration of Indonesian workers in recent years, surging from 72,624 in 2020 to 200,761 in 2022 and 274,964 in 2023 [29,30]. While precise data regarding the number of parents migrating for employment purposes is unavailable, official reports from the Indonesian government indicate that both married individuals, including parents, and unmarried individuals comprise Indonesian migrant workers [29], implying that thousands of children are left behind to be cared for by either one parent or other caregivers. These children may encounter various challenges following parental separation, although empirical evidence on this matter is scarce, with only four studies in the country primarily focusing on the educational benefits derived from remittances [12,31,32], as well as emotional and behavioural issues [33]. 

There is limited evidence and insight into the impact of parental migration on the LBC’s social well-being and risk factors for their mental well-being following separation from their parents [33,34]. This study aimed to address this knowledge gap by qualitatively exploring the perspectives and experiences of LBC in two rural districts in Indonesia regarding how parental migration and the subsequent new life circumstances they encountered have influenced their social and mental well-being. The LBC are part of the family left behind, who directly experience challenging consequences of the separation from their parents. Therefore, understanding their views and lived experiences regarding the social and mental well-being challenges they face could provide valuable insights and evidence for government and non-governmental organisations and institutions to inform policy and practices that address and support LBC needs in Indonesia and beyond.

## 2. Methods

### 2.1. Study Settings

The research was conducted in Belu and Malaka districts, East Nusa Tenggara province, Indonesia. Malaka was formerly part of Belu district before the administrative division in 2012. Both districts share a border with East Timor to the East, with Belu covering a total area of 1284.94 km^2^ and Malaka covering 1160.63 km^2^ [35,36]. Belu district is occupied by a total population of 204,541 people, including 103,619 females and 100,922 males, while Malaka has a total population of 171,079 people, comprising 87,587 females and 83,492 males [35,36]. Administratively, there are 12 sub-districts in each district, 99 villages in Belu, and 127 villages in Malaka [35,36]. Both districts have a similar number of healthcare facilities, with Belu having three hospitals, 17 public health centres, and 21 sub-public health centres, and Malaka having one hospital, 17 community health centres, and 25 sub-community health centres [36,37,38]. The current data show that there were 57,167 Indonesian migrant workers from East Nusa Tenggara province (including Malaka and Belu) working overseas in 2022 [29]. To the best of our knowledge, there have never been any studies exploring in-depth the lived experience of social and mental health consequences of parental migration from the perspectives of the LBC themselves in the context of East Nusa Tenggara, including Belu and Malaka districts, and in Indonesia as a whole [39]. Belu and Malaka districts were selected as the study sites because of the feasibility, familiarity, and potential of undertaking the study successfully.

### 2.2. Study Design and Participant Recruitment

A qualitative study employing in-depth interviews was conducted to explore the views, perceptions, and experiences of adolescents (referred to as LBC) residing in the Belu and Malaka districts of Indonesia regarding parental migration and its impacts on their social and mental well-being. The qualitative approach was chosen as the best means of acquiring authentic accounts of participants’ perspectives in their settings, providing an opportunity to reveal profound insights into their lived experiences [40]. To inform and recruit potential participants, the study information sheets were initially posted on information boards at village offices in the study settings. The information sheets contained brief explanations about the aims of the study, their role in the study if they decided to participate, how the data would be treated and for what purpose the data would be used after the project, and the ethics approvals for the study. Information on these aspects was provided to help the potential participants make their informed decision on whether to participate. Potential participants (the LBC and/or their caregivers) who expressed interest and consented to participate were recruited and interviewed. Initial participants were encouraged to distribute the information to their eligible peers who might wish to participate. The inclusion criteria required participants to be (i) an adolescent with one or both parents migrating for work, (ii) aged between 14 and 18 years, and (iii) willing to participate in the interviews voluntarily. Twenty-four LBC (11 from Belu district and 13 from Malaka) participated in this study. Caregivers of under 18 years old LBC were asked to give consent for the children to participate in this study. They signed and returned informed consent to the field researcher during the recruitment process.

### 2.3. Data Collection

Data collection was conducted using individual face-to-face in-depth interviews. Most interviews were carried out in a private room at the village offices, and a few were conducted in the participant’s houses. Interviews took place at a time the researcher and participants mutually agreed upon. The participants under 18 years of age were offered the opportunity to be accompanied by their mothers or caregivers, but none of them required to do so. Only the researcher and each participant were present in the interview room. Interviews were conducted in Indonesian and recorded using a digital recorder, and field notes were taken during the interviews. The duration of the interviews ranged from approximately 30 to 45 min. Prior to the interviews, participants again received verbal explanation about several aspects of the study, which had been provided in the information sheets during the recruitment process. After the verbal explanation, each of them was again asked whether they understood and firmed their decision to participate. All the participants who met the field researcher on the mutually agreed upon interview days agreed to continue with their decision to participate (see Supplementary Material: Interview Guide). Before commencing the interviews, each participant was required to sign a written consent form to indicate their informed consent to their participation. The recruitment of participants and the interviews were concluded upon the researchers’ perception that the gathered information or data had reached a sufficient level of depth to effectively address the research objective. The determination of data saturation was established by comparing the responses or information provided by a few last participants with those of earlier ones, thus indicating a saturation point in the available data.

### 2.4. Data Analysis

Data analysis began with the verbatim transcription of digitally audio-recorded interviews, during which fieldnotes from each interview were integrated into the actual transcript. The transcripts were comprehensively analysed in Indonesian which also helped maintain the sociocultural meanings attached to the information provided by the participant [41]. For this publication, the selected quotes were translated into English by NKF and ALS who are both fluent in Indonesian and English. To maintain accuracy, checking and rechecking between the original transcript in Indonesian against the translation in English was conducted throughout data analysis and manuscript writing stages (by NKF and ALS) [42]. The English translation was also checked by other authors during the manuscript drafting and revision processes to maintain its quality. A qualitative data analysis framework was used to guide data analysis [43]. The framework offers a systematic approach to managing qualitative data in a coherent and structured way to enhance the rigour, transparency, and validity of the analytic process. 

The approach started with the familiarisation with the data which already began alongside the transcription process through listening to the audio recordings, studying the fieldnotes, and then reading each transcript after transcription, breaking down the data or information into small chunks of data extracts, and making comments or labels to the data extracts. This was followed by the identification of a thematic framework. During the familiarisation with the data, the researchers also started the process of abstraction and conceptualisation by writing down recurrent key issues, concepts, and themes, according to which the data were examined and referenced [43]. In this way, the researchers set up a thematic framework within which the data or codes conducted in the next step were shifted and sorted. The thematic framework was derived from or informed by research questions and aims and the collected data [44]. The identification of the thematic framework was performed simultaneously with the process of coding or indexing the transcripts. The coding process started with open coding by listing all codes that had been made to the data extracts to identify similar or redundant codes and reduce the long list of open codes to a manageable number. This was followed by closed coding where similar codes were grouped under the same themes and sub-themes that had been identified. This also involved changes and refinement of the themes. Data were then charted by arranging appropriate thematic references in a summary chart which enabled comparison across interviews and within each interview. Finally, the data were mapped and interpreted as a whole [43,45].

### 2.5. Ethical Consideration

The study obtained ethics approvals from the Human Research Ethics Committee, Torrens University Australia, Australia (No. 0222) and the Medical and Health Research Ethics Committee, Krida Wacana Christian University, Indonesia (No. SLKE: 1365/SLKE-IM/IKKM/FKIK/KE/X/2022). Before conducting the interviews, participants received comprehensive information regarding the study’s objectives and the potential future use of the data they provided. They were assured of the confidentiality and anonymity of their responses, with all identifying details such as names and addresses being omitted from transcripts to safeguard data anonymity and prevent any potential association of comments with individual participants. Furthermore, participants were informed of the voluntary nature of their involvement and reassured that they could withdraw from the study at any point without facing repercussions. Given the nature of the project and the possibility of emotional discomfort during the interviews, participants were advised that support from a counsellor, arranged by the research team prior to the study, was available free of charge if needed.

## 3. Results

### 3.1. Sociodemographic Characteristics of the Participants

The 24 LBC participating in this study consisted of 21 girls and three boys, aged between 14 and 18 years (see Table 1). Nearly half of them (*n* = 11) had both parents migrating for work and lived in a different household with their grandparents (*n* = 7), aunties (*n* = 3), or older siblings (*n* = 1), while the rest (*n* = 13) had only their fathers migrating for work and lived with their mothers. Most of them attended junior high school (*n* = 15), and a few enrolled in senior high school or recently started university.

The findings were grouped into two main themes, including (i) social well-being of LBC: peers’ negative labelling and changes in familial roles; and (ii) mental well-being challenges among LBC and the associated risk factors: fractured emotional attachment and child–parent disconnection; new life situations and the consequences on the mental health of LBC; and unfulfillment of the LBC’s basic needs and educational needs. A detailed elaboration on the four themes is presented below.

### 3.2. Social Well-Being of LBC: Peers’ Negative Labelling and Changes in Familial Roles

Parental migration for work had consequences for LBC’s relationships with peers and changed familial roles that influenced their social lives, negatively impacting their social well-being. Some LBC across both settings recounted experiences of negative labelling and mistreatment from peers due to parental migration or living without one or both parents. Examples included being taunted, subjected to physical harshness, and ridiculed, all of which had a distressing and frustrating impact on their social well-being. The following story of a girl whose parents migrated to Kalimantan three years ago illustrates such experiences faced by several children who participated in this study:

*P (participant): Sometimes I argue with my friends, and what makes me sad is that some said I am a child with no parents. This made me cry sometimes. Also, they often tease me if I cry, they would say: ‘Who are you going to tell? You don’t have parents here, it’s sad to live without mum and dad’…. So, I often don’t want to mingle with them because of that. Some of them were very close to me but not anymore, they said such things so I keep distance from them*.

(A girl, 14 years old)

The migration of one or both parents to other places can also change familial roles for LBC, negatively impacting their social relationships, lives, and well-being. For instance, both LBC living with their mothers and new non-parental caregivers would find themselves burdened with responsibilities for daily household chores, which limited their ability to engage in social activities with friends after school. Responsibilities such as house cleaning, laundry, cooking, childcare, and tending to livestock became routine tasks, preventing them from participating in extracurricular social interactions with their friends. Such daily routine chores would be usually performed by migrating fathers or both parents and are not daily responsibilities of children in non-migrant families. The experiences of these LBC indicated that the circumstance compelled them to shoulder familial responsibilities, negatively impacting their social well-being and leading to feelings of frustration and stress:

*P: I think there is too much to do at home since my father left several years ago. I have to feed the pigs and chickens in the morning and afternoon. I have to fetch grass for the cow [they have one cow] every afternoon. I also look after my younger siblings at home. I have hardly mingled with my friends since my father left. Sometimes I feel annoyed and stressed, but I have to help my mum*.

(A boy, 17 years old)

*P: I rarely hang out with friends after school or on weekends.*

*R (researcher): Why? Please explain!*

*P: My mum and dad went to Malaysia last year; my two younger siblings and I are living with my grandparents, so after school, I have to look after them and help my grandmother cook and wash clothes.*

*R: You do all that every day, how do you feel?*

*P: Sometimes I feel sad and stressed, not only because I miss my mum and dad but also because I feel burdened and frustrated with all of this. I have to stay home every day, unlike before*.

(A girl, 14 years old)

The participants’ narratives also highlighted the changes in traditional family roles and structures due to parental migration, resulting in shared responsibilities among the children and their mothers or caregivers. Despite the disruption of their social well-being, feeling burdensome, frustrating, and stressful, the children found themselves compelled to take on new tasks and contribute to household duties.

### 3.3. Mental Well-Being Challenges among LBC and the Associated Risk Factors 

#### 3.3.1. Fractured Emotional Bonds and Child–Parent Disconnection

Parents play a crucial role in fostering children’s positive psychological and educational development. Thus, the absence or separation from one or both parents can negatively influence children’s development in these aspects. The stories of all the LBC with one or both parents migrating across the study settings highlighted their experiences of mental health challenges they faced due to the migration of one or both of their parents for employment, either within Indonesia or overseas. The mental health challenges facing them seemed to stem from fractured emotional bonds following the migration of their parents. These manifested in feelings of deep and prolonged sadness, stress, disengagement from daily activities, and a longing for parental presence. The following narrative of a 15-year-old girl reflects the fractured emotional bond she experienced following her father’s migration to Malaysia three years ago:

*R: Could you please tell me a bit more about your experience when your dad left home for Malaysia?*

*P: Oh, I was very sad to see him taking the bus to Kupang [name of provincial city]. I felt hurt, it was painful here [pointing to her chest].*

*R: Why did it make you feel sad and hurt?*

*P: Because it felt like I lost my dad. I was attached to him, always slept next to him every night before he left. I cried a lot for months. I was very sad because suddenly my dad was not in my life. You can imagine what I felt, I was with him every day and night, and suddenly, he was gone. It was very difficult, it hurt my feelings. It took me months to learn to live without my dad at home.*

*R: Now you don’t feel sad or hurt anymore, right?*

*P: Sometimes I still cry when I remember him or see other children accompanied by their dads to school for parental meetings or at the end of the semester. I have been missing him a lot lately.*

Non-involvement of the LBC in discussions with parents regarding parental migration, the unpreparedness of LBC to live without the presence of one or both parents and short notice before the parents’ departure or migration were also contributing factors to the fractured emotional bond or mental health challenges that hurt them. The experiences of such situations are reflected in the story of an 18-year-old girl when her father moved to Papua for work eight years ago:

*R: How did you know that your dad planned to migrate to Papua for work?*

*P: At that time, I didn’t know anything about his plan; I didn’t know that he wanted to go to Papua.*

*R: So, he didn’t tell you at all….*

*P: Dad and mum told me, my brother and my sister a week before he left. It felt very sudden, the three of us immediately cried when we were told about it. What in my head was, ‘Oh, my dad is going to leave us, and we will live without him’. It felt very sad and hurt.*

*R: Could you please tell me more, why did you feel so?*

*P: I felt hurt because I was left by my dad; it was very hard for me. They [his dad and mum] didn’t let us [the children] know about the plan or discuss it with us. I was still very little, I felt like I wasn’t prepared to live without my dad. For some months after he left, I did feel like something was gone from my heart, I felt painful but I didn’t understand it. I guess maybe that is what people call broken heart.*

Furthermore, child–parent communication disconnection following parental migration generated emotional challenges for the LBC. Such disconnection was described by most participants in both settings as intensifying their sense of separation from their parents, leading to a perceived loss of parental affection previously expressed through care, attention, and shared experiences before the migration. The lack of communication tools such as cell phones, along with the unavailability of signals and internet connections in areas where LBC lived or the parents moved into were the main causes of child–parent communication disconnection, which was emotionally challenging for the LBC and influenced their mental well-being:

*R: You said earlier that the connection with your dad and mum was lost, what do you mean?*

*P: After mum and dad left, we lost communication completely. I couldn’t talk to them or see their faces for almost two years because my aunt didn’t have a cell phone, and my mum and dad didn’t have a cell phone either. They said that there were no signals or connections at work location where they first arrived. So, we had no contact, completely disconnected for two years. It felt very painful and unbearable for me. In my heart, I couldn’t accept it. I felt hurt and angry at my mum and dad, but there was nothing I could do.*

*R: Could you elaborate further on what you felt was painful and unbearable?*

*P: Yes, at that time, I felt hurt because not only was our communication cut off, but I also felt there was a loss of parental love. My dad usually bathed me every morning and prepared me for school, mum prepared food, et cetera. After they left, all of these disappeared dramatically, instantly. So, it felt like suddenly there was a distance between us*.

(A girl, 18 years old)

The above narrative highlighted how the disconnection in child–parent communication negatively affected their emotional bonds with their parents and their mental well-being. Considering the lack of professional social and health services to support these LCB in the study settings, it is highly likely that such a situation could have further negative influences on their mental well-being in the future.

#### 3.3.2. New Life Situation and Its Consequences on the Mental Well-Being of the LBC

The migration of one or both parents to other areas within Indonesia or abroad for work significantly affected the daily routines and mental well-being of the LBC. The interviews revealed that the mental health challenges facing these LBC appeared particularly worse among those with both parents migrating compared to the ones with only fathers migrating, as they faced new life circumstances and coped with any possible consequences without any parental support. Transitioning into these new life situations required considerable effort, resilience, and adaptability from the LBC as they adjusted to new routines and lives with non-parental caregivers, such as grandparents, uncles, aunts, or older siblings. These adjustments proved burdensome for them, resulting in various difficulties including sleep disturbances, nighttime awakenings, diminished enthusiasm, inability to concentrate at school, and withdrawal from social interactions. These challenges culminated in experiences of stress, sadness, depression, and anger. The stories of the following boy and girl exemplify the hardships encountered during the initial phase of separation from both parents:

*R: Could you please elaborate further about the situation after your parents left?*

*P: It felt like I suddenly entered a new life without a mum and dad; living with grandparents. At that time, I was only 7 or 8 years old, and I had to face such a situation I was very sad. I still remember that I had difficulty sleeping every night for about six or seven months. I always woke up in the middle of the night, sat up in bed and cried.*

*R: Then what did you do at those times?*

*P: I often stayed in my room, cried, and asked to be taken to my mum and dad. I didn’t talk to anyone in the house for a few months, just kept quiet. It took time for me to adjust to the new living situations with grandma and grandpa. Mum and dad brought me here [to grandparent’s house, from another village where they lived] before they went overseas*.

(A girl, 15 years old)

*P: One of my difficult experiences was that I had to live in a new family, I was left to my aunt, a new family atmosphere. My aunt and her husband had rules in their family that I had to follow; daily routines were burdensome. I often felt stressed, and angry, but I could only cry. It felt really sad at that time*.

(A boy, 18 years old)

Moreover, both parents’ migration presented more significant challenges for the LBC living with non-parental caregivers, not only due to the new circumstances they found themselves in but also because of differences in the attitudes, behaviours, and treatments of their caregivers compared to their parents. For example, some caregivers’ harsh demeanour and disciplinary actions, divergent from those of the LBC’s parents, posed challenges for some of them to navigate. Additionally, the difference in the treatment of caregivers towards the LBC compared to their own children further compounded the situation. These challenges proved difficult for the LBC to handle and adjust to the new circumstances positively, especially considering their young age and limited experience. Consequently, they experienced feelings of hurt, fear, sadness, and difficulty finding joy in their daily lives, which negatively influenced their mental well-being. The stories of two teenage girls, aged 16 and 17, who were left by both parents several years ago, respectively, and resided with their aunt and older sibling, reflect these experiences:

*P: The thing that often makes me feel sad is the attitude and treatment of my aunt and her husband towards me. I feel like they are sometimes rude and very strict with me. They behave like that to me but not to their children. Sometimes, I feel like I’m not part of their family, not fully accepted. It feels different from the attitudes and treatment of my parents towards me. If I think about these, I feel sad and hurt [looked down in tears].*

*P: Living with my oldest brother’s family is very different. I never asked for this and that, I’m afraid of him. If he is angry, it would be very scary. With my mum and dad, I could ask for permission to go and play with friends anytime. He and his wife are very strict. I feel like I don’t enjoy my daily life that much like the other kids. I’m now 17 years old and I feel a bit okay but I would feel much better if mum and dad are here and I live with them.*

#### 3.3.3. Unfulfillment of the LBC’s Basic Needs and Educational Needs

Parental labour migration also precipitated personal and educational challenges for the LBC with one or both parents migrating. Most of LBC across the study settings recounted stories of deprivation or struggles to fulfil basic daily needs. The inability of mothers or caregivers, such as grandparents and aunts, to provide enough food for the entire family and meet the LBC’s needs for clothes, toys, or pocket money for snacks were some instances of difficulties experienced by the LBC. These challenges were recognised in their narratives as contributing factors to the mental health difficulties they encountered, including feelings of pity towards their mothers or caregivers, dissatisfaction, sadness, and discontentment with their living situations, which affected their mental well-being. Witnessing the daily struggles of their mothers or caregivers added to the negative emotions they experienced. The following accounts from two of them, describing their living conditions with their mother and grandparents, exemplify the hardships that contributed to the mental health challenges they faced:

*R: You mentioned earlier about difficulties in your family as one of the reasons you feel sad, can you tell me more?*

*P: I mean, it has been really difficult for grandpa and grandma to provide food for us or fulfil our needs every day. It’s difficult even to buy rice, fish, vegetables, or meat. Sometimes, we just eat cassava.*

*R: Then what about your other needs?*

*P: I don’t really think about new clothes or toys or pocket money for snacks, even though I really want to, but circumstances don’t allow it. I often feel like our fate and circumstances are very unfortunate. I feel sorry for my grandparents because they bear the burden for me and my younger siblings since mum and dad went to Malaysia two years ago*.

(A girl, 14 years old)

*P: Seeing the situation in our family, I am very concerned, I feel sad and sorry for my mum.*

*R: Why? Can you tell me more?*

*P: My mum struggles to the death to take care of all of us [the girl and her siblings]. My dad has been to Timor Leste for two years and hasn’t come back or sent us any money. Sometimes I see mum crying, and that really hurts me. I know she is sad and stressed, I am also stressed. I feel sorry for her. She has to try so hard for us, to provide for our daily needs*.

(A girl, 14 years old)

Furthermore, unmet educational needs played a role in the mental well-being of LBC. The majority of these LBC in both settings shared experiences of being unable to pay school fees or lacking sufficient supplies and uniforms due to their mothers’ or caregivers’ financial constraints. The experience of unmet educational needs, coupled with seeing other children having their needs fulfilled, intensified feelings of sadness and a sense of being disadvantaged in life. For some LBC, this situation also led to feelings of embarrassment and inferiority compared to peers with better living conditions. These circumstances had detrimental effects on their mental well-being, as evidenced by the following quotations:

*P: I still remember, after my dad left us for Malaysia, at the beginning of the new school year, I didn’t have books, pencils, ball pens and new uniforms. I cried and didn’t want to go to school. I felt sad because other friends had new uniforms, but I wore my old uniform, which was too small. My mum couldn’t buy new uniforms, books and pens at that time, because she didn’t have money. I stopped going to school for a year*…...

(A girl, 16 years old)

*P: We are experiencing difficulties in many aspects because grandpa and grandma don’t have paid jobs or monthly incomes. My school fees are often delayed in payment. I often feel very embarrassed because the names of students who haven’t paid are usually posted on the notice board, and other students can read it*.

(A girl, 14 years old)

## 4. Discussion

Parental labour migration has been associated with various negative challenges on the LBC [15,27]. To our knowledge, this study marks the first qualitative exploration of how parental migration and its associated factors affect the social and mental well-being of LBC in Indonesia. Parental labour migration or the absence of one or both parents in LBC’s lives has negative social consequences, including stigma and discrimination against them, which have been associated with increased stress, depression, and suicidal ideation and behaviours among them [46,47,48]. Our findings echo these social consequences, highlighting the negative impact of one or both parents’ migration on LBC’s social well-being reflected in withdrawal from or broken social relationships with peers. Moreover, our findings highlight the burden of responsibilities for household chores and childcare, which limited the LBC’s social activities and interactions, and challenged their social well-being, resulting in sleep disturbances, and diminished enthusiasm in daily life. The findings indicate the need for the establishment of community-led initiatives, such as peer mentoring, and extracurricular or recreational activities, that can create safe and supportive environments for LBC, fostering positive relationships with others within communities and resilience of the LBC [49,50].

Consistent with previous studies [15,51], the current study highlights the adverse challenges of parental labour migration on the mental health well-being of LBC, as evidenced by feelings of emotional detachment, sadness, stress, depression, crying, and self-isolation. Moreover, the study identifies various new risk factors for the mental health challenges faced by these LBC which have been reported in previous findings [14,26], including sudden disconnections of child–parent communication following parental migration, which intensified their feelings of separation and perceived loss of parental affection and support. The lack of LBC’s involvement in discussions regarding parental migration, inadequate preparation for living without parental presence, and short notice before parents’ departure were also significant stressors for mental health challenges faced by the LBC in this study. Parents play vital roles and support for children in regulating emotional responses, dealing with stress, and controlling behaviours by offering encouragement, expressing affection and admiration, and fostering feelings of safety [52,53]. Thus, parental migration and the ensuing disconnection in child–parent communication not only affect the mental health well-being of LBC but also hinder their ability to effectively cope with the parental absence in their lives. Given the low education levels of their parents and the absence of professional support services for children in the study settings, migrant parents may not be fully aware of the potential negative mental health consequences of their migration on their LBC. Therefore, interventions such as mental health counselling are crucial to address emotional distress and enhance coping mechanisms for LBC [50]. The findings also call for intervention programs that facilitate communication channels between the LBC and migrant parents and promote the involvement of children in parental migration-related discussion and decision making, as strategies to prevent the feelings of dramatic or sudden separation from their parents.

Most LBC in Indonesia and some settings are typically under the care of family caregivers after parental migration [54,55], as also observed in this study. What our study adds to the existing knowledge is how transitioning to new life situations with non-parental caregivers and navigating dynamics within the caregivers’ families, including the experience of harsh demeanour and treatment, posed significant challenges to the mental well-being of some LBC with both parents migrating. It can be argued that adjustments to such life transitions and family dynamics require considerable effort and adaptability, especially for young children with limited knowledge, experience, and support [13]. Previous findings have underlined inadequate care and lack of social support for the LBC following the migration of their parents as risk factors for various mental health issues facing them [14,26]. Our findings also highlight the experience of difficulties in fulfilling necessities within both mothers’ and non-parental caregivers’ families and the unmet educational needs of the LBC due to financial constraints facing the caregivers as significant stressors for mental health issues among the LBC. The possible reasons for such financial constraints may include the lack of mothers’ and caregivers’ incomes and remittances by migrant parents. Previous findings on the influence of parental migration on the education of LBC have been inconsistent with some reporting negative impact on LBC’s academic performance due to parental absence [56,57,58], while others reported positive contributions to LBC’s education enrolment and attainment due to parental financial support or remittance [11,12]. Additionally, the current study highlights the exposure to daily struggles of mothers or caregivers to fulfil necessities as influencing factors for the LBC’s mental health. Our findings have implications for the government of Indonesia to improve its policies on social support and ensure that its existing social support or programs for families, including non-cash food assistance, family hope program, direct cash assistance, food risk mitigation, and rice social assistance, cover families caring for LBC [59,60], which may contribute to positive development for LBC under their care. 

### Limitations and Strengths of the Study

The study involved a small number of LBC in two rural districts in Indonesia, thereby reflecting their specific perspectives and experiences that may differ from those of LBC in other settings with distinct characteristics. The use of the snowballing sampling technique might also have introduced recruitment bias, as most participants appeared to be acquainted with each other. Consequently, LBC who were not part of the same networks as the current participants, and thus may have had different experiences, might not have been covered in this study. Therefore, the findings reported in this study do not necessarily reflect the full picture of the perceptions and experiences of LBC in the study settings. As is the case of many other qualitative studies, the findings of this study are not meant to be generalised to other LBC in urban districts or different settings in Indonesia and beyond. Moreover, this sampling technique led to the recruitment of more girls than boys who may have different stories. Nonetheless, the strength of the study is that, to the best of our knowledge, it was the first qualitative research in Indonesia to delve into the personal views and lived experiences of LBC regarding the impact of parental labour migration on their social and mental well-being. As such, the findings offer valuable insights to guide the development of policies and intervention programs aimed at addressing the needs of both LBC and their caregivers or families within the study settings and beyond.

## 5. Conclusions

The study presents the significant impact of labour migration on the social and mental health well-being of LBC in rural districts in Indonesia. The engagement in family responsibilities which limited social activities and interactions, and adverse social repercussions associated with parental migration emerged as significant factors affecting the social well-being of LBC. The separation from one or both parents led to fractured emotional bonds among the children, resulting in a spectrum of negative emotions including stress, anxiety, sadness, depression, frustration, loss of motivation, and self-imposed isolation, which affected the mental well-being of these children. Factors such as abrupt breakdowns in parent–child communication, transition to new life situations with caregivers, the dynamics within caregivers’ family, and unmet basic and educational needs, were also significant contributors to the mental health challenges encountered by LBC. Future large-scale studies are recommended to investigate the diverse needs of both caregivers and LBC, aiming to foster a more comprehensive understanding of their needs and inform the development of tailored and effective interventions and support services.

## Figures and Tables

**Table 1:** Sociodemographic profile of the participant.

No	Sex	Age	Education	Living with	Parents Who Migrated	Country/Place of Migration
1	Female	14	Junior High School student	Grandparents	Both parents	Kalimantan
2	Female	18	Senior High School student	Mother	Father	Malaysia
3	Female	17	Senior High School student	Older sibling	Both parents	Malaysia
4	Male	18	University student	Aunty	Both parents	Malaysia
5	Female	18	University student	Mother	Father	Papua
6	Female	18	University student	Mother	Father	Kalimantan
7	Female	18	University student	Mother	Father	Papua
8	Male	18	University student	Mother	Father	East Timor
9	Female	18	Senior High School graduate	Aunty	Both parents	Papua
10	Male	17	Senior High School student	Mother	Father	Bogor
11	Female	15	Junior High School student	Grandparents	Both parents	Kalimantan
12	Female	14	Junior High School student	Grandparents	Both parents	Kalimantan
13	Female	14	Junior High School student	Grandparents	Both parents	Kalimantan
14	Female	14	Junior High School student	Grandparents	Both parents	Malaysia
15	Female	15	Junior High School student	Mother	Father	Malaysia
16	Female	14	Junior High School student	Grandparents	Both parents	Malaysia
17	Female	16	Junior High School student	Mother	Father	Malaysia
18	Female	14	Junior High School student	Mother	Father	Kalimantan
19	Female	14	Junior High School student	Mother	Father	East Timor
20	Female	14	Junior High School student	Mother	Father	Malaysia
21	Female	15	Junior High School student	Mother	Father	Papua
22	Female	16	Junior High School student	Aunty	Both parents	Papua
23	Female	15	Junior High School student	Grandparents	Both parents	East Timor
24	Female	14	Junior High School student	Grandparents	Both parents	Kalimantan

## Data Availability

The data presented in this study are available on request from the corresponding author. The data are not publicly available due to restrictions set by the human research ethics committee.

## Supplementary Materials

The following supporting information can be downloaded at: https://www.mdpi.com/article/10.3390/ijerph21060793/s1, File S1: Interview Guide: Children with One or Both Parents Migrating.
