# Supplementary material for: Parental Migration and the Social and Mental Well-Being Challenges among Indonesian Left-Behind Children: A Qualitative Study
Source: Int J Environ Res Public Health. 2024 Jun 18;21(6):793. doi: 10.3390/ijerph21060793 (PMC11203627; doi:10.3390/ijerph21060793)
Supplement: Supplementary file 1 [file ijerph-21-00793-s001.zip › ijerph-3007529-supplementary.pdf]

TORRENS UNIVERSITY AUSTRALIA

Interview guide: Children with one or both parents migrating

Date: ________________________________(Date)
Interviewer:____________________(Name)

Greeting......

Thank you very much for agreeing to participate in this interview, and thanks for your time, I do appreciateit.

Is it OK if I record the interview and take notes during the interview?

When I write up the results of this study, I will remove all personal information to ensure that the information or comments you provide in this interview remain anonymous. Is this OK with you? You can withdraw your participation or ask me to stop this interview if you do not want to continue or feel uncomfortable during this interview without any consequences.

As you have known through the study information sheet and our initial conversation once you confirmed your willingness to participate, the purpose of this study is to gain your insights into parental migration- related mental health and social challenges you face, the associated factors and the impact of mental health challengeson the children.

Demographic information

Age :
Sex :

What is the highest education you received? : Not at all/primary/secondary/tertiary

Who migrated for work : father/mother/both

Please explain what did you feel/ challenges you faced when your parent(s) left

home?

- Sad, stressed, angry, lonely, depressed, etc, why?
- Scare and worried about yourself or your parents? Why? Please explain more.
    - Are those feelings gone or do you still feel them sometimes up to now? Why? Tell me more about it.

How often do you talk to your parent(s) since they left?

- How do you feel about it?
- Does it have an influence on you emotionally/psychologically? How? Please explain more about it.

Please explain how you feel about the separation from your parents?

- Do you feel there is a disconnection/distance between you and your parent(s) since they left? Why? Please explain more about it.
- Do you think it influences your life? How or in what way? Please explain more about it.

Who do you live with since your parent(s) left?

- Grandparents, uncle, aunt, siblings?
- How do you feel that suddenly you have to live with your grandparents/aunt/uncle/siblings? Pleaseexplain more about it.
    - How do they treat you: behaviours/attitudes? Do you feel fine with that or not? Why? Please explain more.
    - Do you think their attitudes/behaviours or the way they treat you have an impact on youremotional/psychological condition? How? Please explain more about these.
- What are the works or activities that you are assigned to do regularly? Please explain.
    - How do you feel about them? Feel burdened, pressured, etc, why? Please explain.
- What is the living condition of your grandparents/uncle/aunt/siblings' family you livewith?
    - Are your needs (food, clothes, etc) fulfilled or not? Why? Please explain more about it.
    - How do you feel about that? Please explain.

What about your school/education-related needs, are they fulfilled?

- Your school fees, uniform, stationary, etc. Please explain more about these.
- In case, you experience difficulties in fulfilling your educational needs,
    - how do feel about it? .... Please explain
- Do those kinds of feelings you experience influence the way you look at your futureeducation? Why? Tell me more about it.
    - Influence your attitudes and thoughts towards education? Please explain more about these.

Please tell me about your social life?

- Are you often socialised with your friends: playing or hanging out with friends afterschool or during the weekend,
    - If not, why?
    - How does it influence you emotionally/psychologically? Please explainmore

- Are you involved in extra-curricular activities: scouting practices, choir exercises, etc
    - If not, why? Please explain more about these.
- How does it influence you emotionally/psychologically? Please explain more

What about your relationships with your friends?

- Do you have good relationships with many friends or not? Please tell me more about it.
- Have you ever experienced negative attitudes or treatment from your friends dueto your parent(s) being migrant workers in other places/countries? Please explain

Is there anything else you'd like to add?

Could you suggest (distribute information sheet) any friends of yours who might be willing to participate in this research?

Pertanyaan penuntun wawancara: Anak-anak berusia 15-18 yang salah satu atau kedua orangtunyaadalah pekerja migran

Tanggal : ________________________

Pewawancara : ________________________(Nama)

Sapaan awal.....

Terima kasih banyak telah setuju untuk berpartisipasi dalam wawancara ini, dan terima kasih atas waktuAnda, saya sangat menghargainya.

Torrens University Australia Ltd, ABN 99 154 937 005, RTO41343, CRICOS 03389E

Apakah tidak apa-apa kalau saya merekam dan mencatat selama wawancara berlangsung?

Ketika saya menulis hasil dari riset ini, saya akan mengeluarkan semua informan yang menyangkut identitaspribadi untuk memastikan bahwa informsi yang Anda sampaikan dalam wawancara ini bersifat anonim. Apakah hal ini tidak apa-apa menurut anda? Anda bisa menghentikan partisipasi anda atau meminta agar wawancara ini dihentikan bila anda tidak ingin melanjutkan wawancara atau merasa tidak nyaman selama wawancara tanpa ada konsekuensi apapun.

Sebagaimana anda telah baca melalui lembaran informasi dan pembicaraan kita terdahulu ketika anda mengkonfirmasi keinginan anda untuk berpatisipasi, tujuan dari riset ini adalah untuk menggali pemahaman anda mengenai tantangan kesehatan mental yang Anda hadapi setelah orang tua anda bermigrasi, faktor- faktor yang turut berkontribusi terhadap tantangan kesehatan mental yang anda alami dan apa dampak tantangan kesehatan mental tersebut bagi hidup Anda.

Information demografis

Age :

Jenis kelamin :

Tingkat Pendidikan : Tidak sekolah/SD/SMP/SMA/Kuliah

Siapa yang bermigrasi : ayah/ibu/keduanya

Tolong jelaskan apa yang Anda rasakan/tantangan kesehatan mental yang Anda hadapi

ketika orang tua Anda meninggalkan rumah?

- Sedih, stres, marah, kesepian, depresi, dll, mengapa?
- Takut dan khawatir tentang diri Anda atau orang tua Anda? Mengapa? Tolong jelaskan lebih lanjut.
    - Apakah perasaan tersebut sudah hilang atau masih dirasakan sampai sekarang? Mengapa? Ceritakan lebih banyak tentangnya.

Seberapa sering kamu dihubungi orang tua Anda dan berbicara dengan mereka sejak mereka pergi?

- Bagaimana perasaan Anda tentang hal itu?
- Apakah itu berpengaruh pada Anda secara emosional/psikologis? Bagaimana? Tolong jelaskan lebih lanjut tentang itu.

Tolong jelaskan bagaimana perasaan Anda tentang perpisahan dari orang tua Anda selama berbulan-bulan/tahun?

- Apakah Anda merasa ada jarak antara Anda dan orang tua sejak mereka pergi? Mengapa? Tolong jelaskan lebih lanjut tentang itu.
- Apakah menurut Anda hal itu memengaruhi hidup Anda? Tolong jelaskan lebih lanjut tentang bagaimana pengaruhnya terhadap Anda?

Dengan siapa kamu tinggal sejak orang tuamu pergi?

- Kakek, paman, bibi, saudara?
- Bagaimana perasaan Anda bahwa tiba-tiba Anda harus tinggal bersama kakek/nenek/bibi/paman/adik? Tolong jelaskan lebih lanjut tentang itu.
    - Bagaimana mereka memperlakukan Anda: perilaku/sikap? Apakah Anda merasa baik-baik saja dengan itu atau tidak? Mengapa? Tolong jelaskan lebih lanjut.
    - Apakah menurut Anda sikap/perilaku mereka atau cara mereka memperlakukan Anda berdampak pada kondisi emosional/psikologis Anda? Bagaimana? Tolong jelaskan lebih lanjut tentang ini.
- Pekerjaan atau kegiatan apa yang ditugaskan untuk Anda lakukan secara setiap hari oleh opa/oma, paman, bibi atau kakak? Tolong jelaskan.
    - Bagaimana perasaan Anda tentang mereka? Merasa terbebani, tertekan, dll, mengapa? Tolong jelaskan.
- Bagaimana kondisi kehidupan keluarga kakek/nenek/paman/bibi/kakak yangtinggal bersama Anda?
    - Apakah kebutuhan Anda (makanan, pakaian, dll) terpenuhi atau tidak?Mengapa? Tolong jelaskan lebih lanjut tentang itu.
    - Bagaimana perasaan Anda tentang itu? Tolong jelaskan.

Bagaimana dengan kebutuhan sekolah/pendidikan Anda, apakah sudah terpenuhi?

- Biaya sekolah, seragam, alat tulis, dll. Tolong jelaskan lebih lanjut tentang ini.
- Jika Anda mengalami kesulitan dalam memenuhi kebutuhan pendidikan Anda, bagaimana perasaanAnda?
- Apakah perasaan seperti itu yang Anda alami memengaruhi cara Anda memandang Pendidikan atau masa depan Anda? Mengapa? Ceritakan lebih banyak tentangnya.
    - Mempengaruhi performa akademik Anda?
    - Mempengaruhi sikap dan pemikiran Anda terhadap pendidikan? Tolong jelaskan lebihlanjut tentang ini.
- Bagaimana perasaan atau tantangan kesehatan mental yang Anda hadapi, apakah memengaruhipemikiran Anda tentang masa depan Anda? Tolong jelaskan lebih lanjut tentang itu.
- Apa pendapat Anda tentang masa depan Anda?
    - Masih memiliki motivasi yang kuat untuk dapat pekerjaan yang baik di masa depan atau tidak?Tolong beritahu saya tentang hal itu.

Tolong ceritakan tentang kebutuhan sosial Anda, apakah terpenuhi?

- Apakah Anda sering bersosialisasi dengan teman-teman Anda: bermain atau bergaul dengan teman-teman setelah sekolah atau saat akhir pekan?
    - Jika tidak, mengapa?
    - Bagaimana pengaruhnya terhadap Anda secara emosional/psikologis? Tolong jelaskan lebih lanjut
- Apakah Anda terlibat dalam kegiatan ekstra kurikuler: latihan pramuka, latihan Paduan suara, dll?
    - Jika tidak, mengapa? Tolong jelaskan lebih lanjut tentang ini.
    - Bagaimana pengaruhnya terhadap Anda secara emosional/psikologis? Tolong jelaskan lebih lanjut

Bagaimana dengan hubungan Anda dengan teman-teman Anda?

- Apakah Anda memiliki hubungan yang baik dengan banyak teman atau tidak? Tolongceritakn lebih tentang hal ini.

- Apakah Anda pernah mengalami sikap atau perlakuan negatif dari teman Anda karena orang tua Anda bekerja sebagai TKI di tempat/negara lain?
    - Mengapa? Tolong jelaskan lebih lanjut tentang ini.
    - Bagaimana perasaan Anda tentang sikap atau perilaku mereka terhadap Anda? Tolong jelaskan.

Apakah ada hal lain yang ingin Anda tambahkan?

Apakah Anda dapat menyarankan temam-teman Anda yang mungkin bersedia untuk berpartisipasi dalam penelitian ini?
